# Advances in Barrier Membranes for Guided Bone Regeneration Techniques

**DOI:** 10.3389/fbioe.2022.921576

**Published:** 2022-06-22

**Authors:** Ze Yang, Chang Wu, Huixin Shi, Xinyu Luo, Hui Sun, Qiang Wang, Dan Zhang

**Affiliations:** ^1^ Liaoning Provincial Key Laboratory of Oral Diseases, School and Hospital of Stomatology, China Medical University, Shenyang, China; ^2^ Department of Plastic Surgery, The First Affiliated Hospital of China Medical University, Shenyang, China

**Keywords:** guided bone regeneration, barrier membrane, polymer, functional membrane, absorbable material

## Abstract

Guided bone regeneration (GBR) is a widely used technique for alveolar bone augmentation. Among all the principal elements, barrier membrane is recognized as the key to the success of GBR. Ideal barrier membrane should have satisfactory biological and mechanical properties. According to their composition, barrier membranes can be divided into polymer membranes and non-polymer membranes. Polymer barrier membranes have become a research hotspot not only because they can control the physical and chemical characteristics of the membranes by regulating the synthesis conditions but also because their prices are relatively low. Still now the bone augment effect of barrier membrane used in clinical practice is more dependent on the body’s own growth potential and the osteogenic effect is difficult to predict. Therefore, scholars have carried out many researches to explore new barrier membranes in order to improve the success rate of bone enhancement. The aim of this study is to collect and compare recent studies on optimizing barrier membranes. The characteristics and research progress of different types of barrier membranes were also discussed in detail.

## 1 Introduction

Alveolar bone defect is a common oral disease. Insufficient alveolar bone caused by trauma, tumor, periodontitis, and long-time tooth absence has gradually become a huge challenge for the medical application of subsequent implantation, orthodontic, periodontal and functional repair treatments. The absence of natural teeth will cause the loss of functional stimulation of alveolar bone, resulting in progressive, cumulative and irreversible bone resorption, and the alveolar bone cannot maintain the bone contour, nor can it obtain the effect of mucosal support (Cawood and Howell, 1988). There are many ways to improve the alveolar bone, including guided bone regeneration, cleavage of the alveolar crest, bone compression, maxillary sinus lifting, distraction osteogenesis and autologous mass bone grafting (Fu and Wang, 2011; Mohan et al., 2015; Danesh-Sani et al., 2016; Sakkas et al., 2017; Rachmiel et al., 2018). At present, GBR technology is recommended to be used before or during the same period of dental implantation to expand and retain alveolar bone. It has become a widely recognized method for repairing alveolar ridge defects and is the most common bone augmentation technology with the longest clinical application time. After 8 years of follow-up, Kim et al. (2020) found that the success rate of implants followed by GBR treatment was about 77.8%. GBR technology expands the indications of oral implantation, guarantees the biological, aesthetic effects after implantation and restoration as well as reduces the incidence of complications.

**FIGURE 1:**
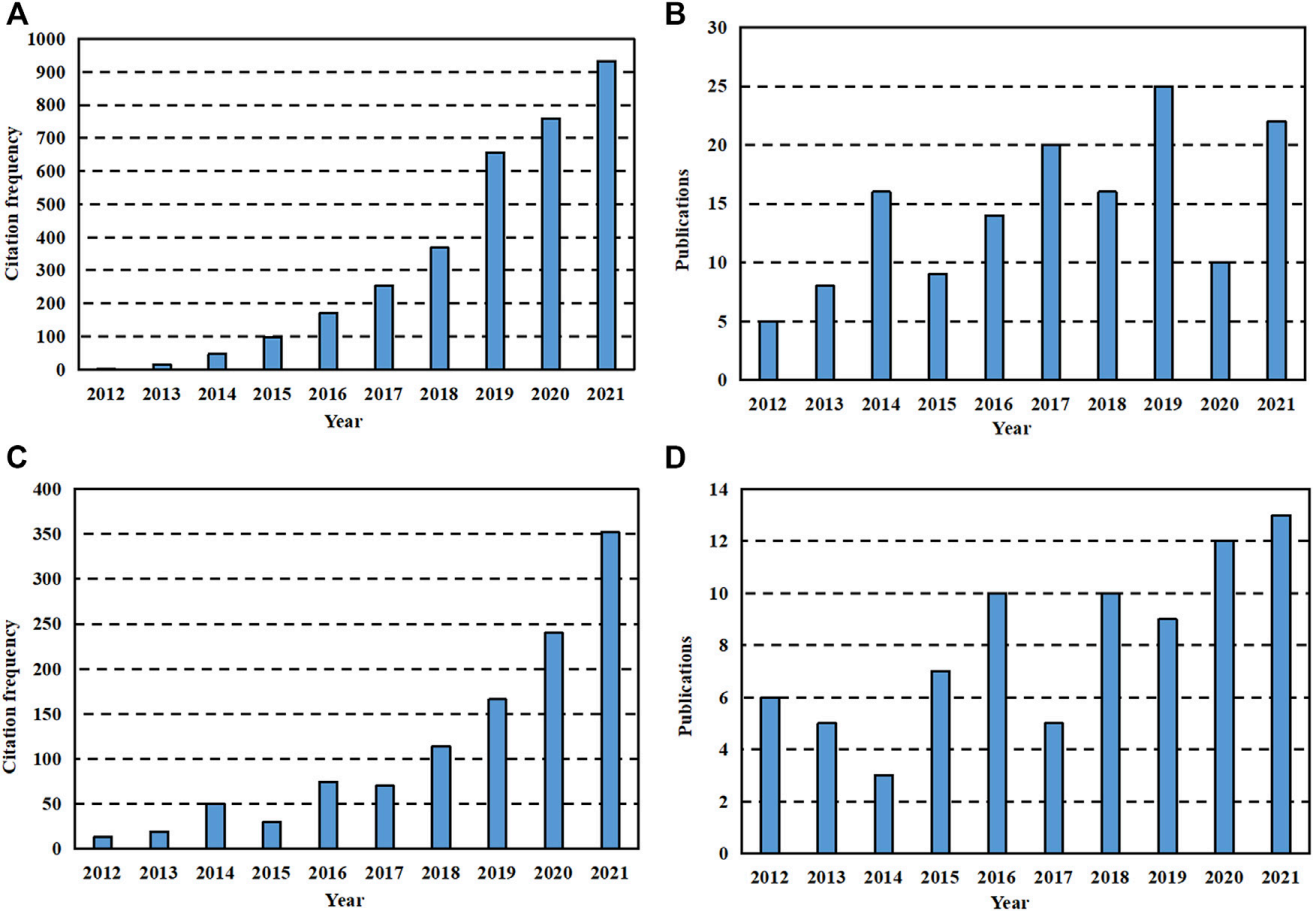
Data search results for last 10 years. **(A)** The citation frequency of literature with “GBR” and “polymer” as keywords retrieved on Web of Science in recent 10 years. **(B)** The publication of literature with “GBR” and “polymer” as keywords searched on Web of Science in recent 10 years. **(C)** The sum of citation frequency of literature retrieved on Web of Science with “GBR” and “ceramic” as one group of keywords as well as “GBR “and “metal” as another group of keywords in recent 10 years. **(D)** The sum of the publication of literature retrieved with “GBR” and “ceramic” as one set of keywords and “GBR “and “metal” as another set of keywords on Web of Science in recent 10 years.

It is well-known that the essential substances in the composition of animals and plants, such as proteins and cellulose, are polymer compounds. The ubiquity in the biological community determines their special status in the field of medicine, so they are most commonly used as medical materials. Biomedical polymer materials have the advantages of designable structure, good biological activity, stable physical properties, wide sources and low price. Therefore, as shown in Figure 1, polymers are always hot spots in the development of GBR applications and show an increasing trend year by year. However, it is worth noting that polymers should have high polymer purity, clean production environment and little residue of polymerization additives due to the presence of monomer impurities. Besides, chemical and mechanical properties shall meet the requirements of medical design and function. Such as hardness, elasticity, mechanical strength and fatigue strength. Finally, the material needs to be compatible with other materials like human tissues so that the implanted material has no side effect on body fluids for long-time use. According to their different uses in the medical field, they can be divided into five categories: 1) Materials that are not in direct contact with the tissues of the organism. 2) Materials in contact with skin and mucous membranes (Goodier et al., 2018). 3) Materials in short-term contact with human tissues (Liu et al., 2018). 4) Materials implanted in the body for a long time (Williams, 2008). 5) Pharmaceutical polymers (Ekladious et al., 2019).

## 2 Performance Requirements of Barrier Membranes in Guided Bone Regeneration Techniques

The principle of guided bone regeneration comes from guided tissue regeneration (GTR) in periodontium. The periodontal soft tissues such as epithelial cells and connective tissues proliferate and migrate relatively rapidly. GBR aims to insulate the soft tissues from the bone defect with a barrier membrane to provide a fairly closed environment for tissue growth. The cells with regenerative ability in the bone defect area proliferate and differentiate to the maximum extent to ensure the priority of osteogenesis and promote the formation of new bone (Elgali et al., 2017; Nowwarote et al., 2018). Selection of appropriate barrier membrane is the deciding factor in the success of guided bone regeneration. As seen in Table 1, ideal GBR barrier membrane should have good mechanical properties, including favorable space-making properties and clinical operability, favorable biological properties such as satisfactory biocompatibility, bioactive properties, tissue selectivity and antibacterial properties. How to better improve the peri-implant bone volume and further increase the degree of osseointegration is one of the most important properties for GBR.

**TABLE 1:** Properties of ideal barrier membrane.

Properties	Purpose	Influencing Factor	Effect	References
Space-making properties	Provide a suitable space in which the regeneration of bone can take place	Plasticity	Adaptation to the bone defect	
		Stiffness	Withstand the compression of the soft tissue	
		Resistance of tear	Withstand the ambient pressure	Elgali et al. (2017)
		Thickness	Thicker: reduce soft tissue ingrowth	Benic et al. (2019)
		Stability of implant	Maintain the defect space	Moses et al. (2008)
		Implant site	Appropriate and effective	Hoornaert et al. (2016)
		Implant site	Steadfast and effective	
		Hold time	Meet the needs of periodontal tissue regeneration, 4∼6w	
			Meet the needs of bone tissue regeneration, 16∼24w	
Clinical operability	Specific requirements conducive to surgical operation	Chemical properties	Cover the bone defect	
			Fit adjacent bone surface	
		Physical properties	Hard: cause the soft tissue cracking	Won et al. (2016), Aprile et al. (2020)
Biocompatibility	Regeneration of tissue	—	Osteopromotive	Won et al. (2016), Rakhmatia et al. (2013)
Bioactive properties	Positive effect on the regeneration of the bone defect	—	Membranes without this characteristic at present	Hoornaert et al. (2016)
Tissue selectivity	Promote bone regeneration and prevent the ingrowth of connective tissue	Porosity	Inhibition of soft tissue, promote bone tissue	Murphy et al. (2000), Sheikh et al. (2017a)
		Osteoconductivity	Allow osteogenitor cells to form new bone tissues	Abdelaziz et al. (2020), Wang and Boyapati (2006)
Antibacterial properties	Resistance to the bacterial invasion	—	Minimize the negative effects of exposure	Annibali et al. (2012)

### 2.1 Space-Making Properties and Clinical Operability

Spatial maintenance of clinical GBR technology requires attention to maintain osteogenic space, so the barrier membrane materials need to have a series of corresponding characteristics to ensure the space maintenance effect (Won et al., 2016). Firstly, it is required to have appropriate plasticity, which can provide a specific spatial structure to promote the functional reconstruction of alveolar bone according to different defect areas. In addition, the barrier membrane needs to meet a specific strength requirement to withstand external pressure, so adequate rigidity and tear resistance are essential. Meanwhile, the thickness of the barrier membrane is different, which affects the space maintenance characteristics during the implantation process. The practice has shown that placing a thicker collagen membrane can reduce the ingrown soft tissue and promote bone formation (Elgali et al., 2017). In practice, whether the stability of the graft can be maintained determines the success of GBR to a certain extent. Therefore, minimizing the movement of the barrier membrane in the operative area can effectively maintain a stable three-dimensional reconstruction spatial structure. In addition, the appropriately placement of barrier membrane and the bone meal implantation will also affect the effect of bone increment after bone grafting (Benic et al., 2019). The barrier membrane applied to GBR plays a crucial role in forming new bone tissue at the implantation site. Some scholars believe that in GBR technology, the barrier membrane is required to last 4–6 weeks, which is the requirement of regeneration time of total periodontal tissue (Moses et al., 2008). Some scholars also suggest that ideal GBR membrane should maintain its barrier function for 16–24 weeks to meet the requirement of bone tissue regeneration time (Hoornaert et al., 2016).

From the perspective of clinical practicability, GBR barrier membrane needs to meet the specific requirements of convenient for surgical operation. The barrier membrane needs to be easy to cover the bone defect area and conform to the adjacent bone surface. In terms of physical properties, if the barrier membrane is too hard, it may interfere with tissue integration in the bone defect area or lead to soft tissue dehiscence (Won et al., 2016; Aprile et al., 2020).

### 2.2 Biocompatibility and Bioactive Properties

Barrier membrane for GBR needs to have good biocompatibility to achieve the effect of supporting tissue regeneration. Good biocompatibility requires that it has no negative effects on the prognosis of peripheral cell tissue, bone defect area, and the patients’ overall health (Rakhmatia et al., 2013; Won et al., 2016). The interaction between barrier membrane and tissue has a positive effect on the surrounding tissue, which further leads to the healing of the defect. If the barrier membrane is absorbable, it should have the ability to degrade or integrate into host tissues, reducing the potential incompatibility caused by the barrier membrane.

Bioactivity refers to the ability of biomaterials to produce chemical bonding with living bone which is an important indicator to measure biomaterials. In the application of GBR, it is mainly reflected in the osteogenic capacity of barrier membranes. First, the barrier membrane itself can evoke a local environment in the defect, which is conducive to the regeneration and differentiation of osteoblasts. The environment created by the membrane is also conducive to the formation and reconstruction of the molecular mechanism of coupled bone in the submembrane defect (Omar et al., 2019). In addition, membrane bioactivity can be improved by designing the membrane structure (Liu et al., 2021). In recent years, it has also become an important way to enhance the osteoinductive ability of membranes by adding some inorganic particles, growth factors, etc. Common additives *in vitro* experiments are Sr-CaP nanoparticles (Ye et al., 2019), octacalcium phosphate (OCP) (Wang Y. et al., 2019), bone morphogenetic protein (BMP) (Yin et al., 2017a) and so on. Common additives *in vivo* experiments are metformin (Met) (Ebrahimi et al., 2021), epigallocatechin-3-gallate (EGCG) (Chu et al., 2019), etc. Barrier membranes will not only have a passive role but can play an active role at the site of bone defect regeneration (Hoornaert et al., 2016).

### 2.3 Tissue Selectivity

One of the critical points of the tissue-selective GBR technique is to promote the regeneration of bone tissue in the bone defect area while preventing the peripheral junction of tissue growth. This requires the relevant performance of the barrier membrane to be guaranteed. In terms of selective tissue growth, the barrier membrane should allow the passage of oxygen, tissue fluid and related bioactive substances but prevent the growth of connective tissue cells and epithelial cells into the defect area. In addition, a certain porosity is required to allow cells to adapt to their surroundings, and to provide cells with fully permeate nutrients (Murphy et al., 2000). Appropriate pore size can inhibit the growth of soft tissue and facilitate the diffusion of substances beneficial to the growth of bone tissue (Sheikh et al., 2017a). The selective healing process of the tissue defect can be achieved in two general directions. One is to promote growth which stimulates the growth of the tissue around the bone defect area. The other is to prevent growth which prevents the growth and implantation of epithelial connective tissue cells (Iviglia et al., 2019).

Tissue integration between the barrier membrane and the adjacent bone contour ensures effective adsorption to grow, contributing to the relative seal between the natural bone and the implant material. Tissue integration accelerates the wound healing process in the surgical area and helps prevent the integration of non-osteogenic components such as fibrous connective tissue into the defect site. The ability to create a membrane space of the material is key to achieving tissue integration. Abdelaziz et al. (2020) proposed that the barrier membrane also needed to have good osteoconductivity. That is to say, the compatibility of the old barrier membrane or scaffold with osteoclasts allows osteogenic cells to grow from the edge of the existing bone to form a new bone tissue structure and achieve tissue integration of bone. GBR surgery requires good soft tissue sealing and long-term wound stability to protect the regenerative process (Wang and Boyapati, 2006).

### 2.4 Antibacterial Properties

Antibacterial property is also a heated topic in the study of barrier membrane modification by scholars nowadays. Bacterial infection has been a common headache in our daily life or clinics. Bacterial adhere on the surface of medical materials, resulting in infections or even failure of materials or surgery operation (Behzadi et al., 2017; Jensen et al., 2017). It has been verified that the adverse effects of barrier membrane exposure on surrounding tissues are mainly due to bacterial invasion. Inflammatory response caused by bacterial invasion can inhibit the growth of osteoblasts, thus affecting the effect of guided bone regeneration and even leading to the failure of surgery. Researchers hoped to ensure or improve bone regeneration by preventing bacterial invasion (Cao et al., 2019). One of the main challenges of GBR restoration is bacterial colonization on the membrane, constitutes to premature membrane degradation (Saarani et al., 2017). Therefore, timely treatment once membrane exposure occurs during the GBR process can minimize the negative effects of exposure (Annibali et al., 2012), which also reflects that tissue integration plays an important role in the success of GBR. Non-resorbable membranes with different pore sizes have been used for exposure experiments. It was found that the osteogenic effect of non-resorbable membranes with pore sizes smaller than the general diameter of bacteria had not been significantly reduced even if they were exposed (Barboza et al., 2010).

## 3 Specific Types of Barrier Membranes

**FIGURE 2:**
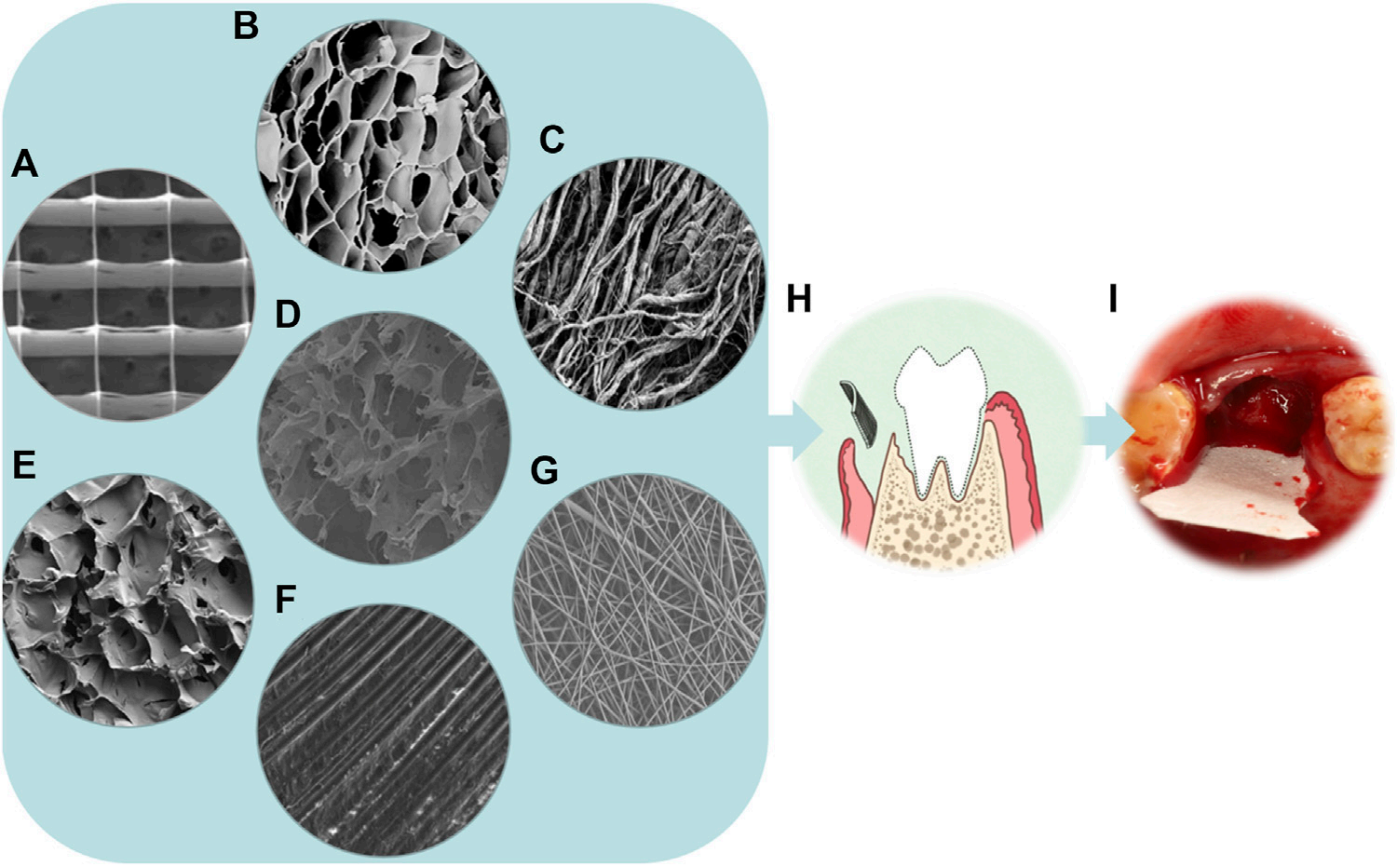
From microscopy to reality of barrier membranes. **(A)** SEM micrograph of the PCL scaffold with the spacing of 500 µm (Dubey et al., 2020). **(B)** SEM micrograph of the γ-PGA/BC composite hydrogel (Dou et al., 2021). **(C)** SEM image of a rougher bottom layer with collagen strands of Bio-Gide (magnification×1,000) (Wu et al., 2018). **(D)** SEM image showing the morphology of the loose layer of an asymmetric porous chitosan membrane (magnification×2,000) (Ma et al., 2016). **(E)** SEM image showing the morphology of the loose cross-linked collagen layer of the aspirin-loaded chitosan nanoparticles contained in collagen-chitosan membrane (ACS-CCM) (Zhang et al., 2017). **(F)** SEM image of the dPTFE membrane (magnification×500) (Korzinskas et al., 2018). **(G)** Field emission scanning electron microscopy(FE-SEM) images of PLGA/PCL electrospinning membranes (magnification×1,000) (Qian et al., 2016). **(H)** Schematic illustration of the principle of GBR. **(I)** The implant placement procedure for BioGuide membrane.

**FIGURE 3:**
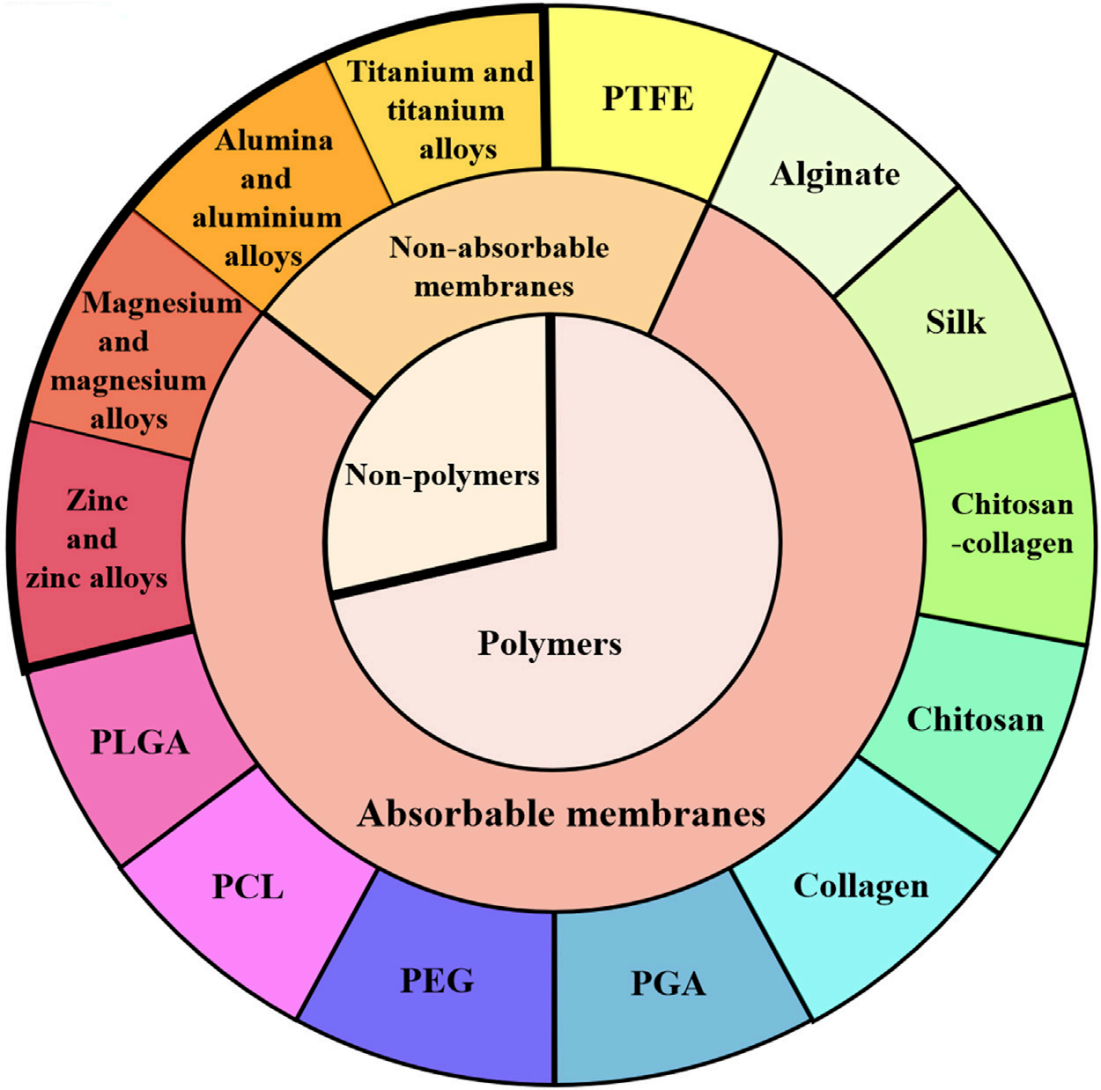
Classification of barrier membranes. GBR barrier membranes are classified from polymer and non-polymer, absorbable and non-absorbable barrier membranes. Several typical barrier membranes in each classification are introduced in the text of this paper.

As shown in Figure 2, different types of barrier membranes can be applied to bone defects during the GBR procedure to play a certain role in osteogenesis. As shown in Figure 3, barrier membranes can be divided into two categories according to the composition, polymer membranes and non-polymer membranes. According to whether it is degradable or not, barrier membrane can be divided into absorbable membrane and non-absorbable membrane. Non-absorbable membranes are still in a minority of the current barrier membrane studies. Judging from the mechanical properties of several common barrier membranes in Table 2, single-component polymers tend to have lower mechanical properties than composite polymers. Both mechanical and biological properties can affect the stability and effectiveness of the barrier membranes. Based on the biological characteristics of several common barrier membranes listed in Table 3, the barrier membranes meet the requirements for the cytotoxicity of surgical implant materials (grade 0–1). Among the rest, PLGA and collagen have class 1 cytotoxicity but are considered biosafe. Their inflammatory responses were within the acceptable range. In addition, it is worth noting that chitosan inflammatory responses are correlated with molecular weight.

**TABLE 2:** Mechanical properties of barrier membranes.

Material	Processing method	Elastic modulus(MPa)	Tensile strength (MPa)	Elongation at break (%)	References
PLGA	Electrospinning	—	2.90(0.31)	—	Jazayeri et al. (2017), Zhao et al. (2008); Fu et al. (2017)
PCL	Solvent casting technique	26.32	19.84	627.58	Tsai et al. (2019), Lee et al. (2018)
PCL/Chitosan	Coaxial electrospinning	13.26 ± 2.79	4.23 ± 0.51	—	He et al. (2017), Shi et al. (2017)
Collagen	Bio-Gide is composed of porcine type I and type III collagen fibers	—	3.4–11.4	9.6–46.8	Elgali et al. (2017), Sheikh et al. (2016), Tolstunov et al. (2019), Ha et al. (2014), Taguchi et al. (2005), Bozkurt et al. (2014), Ortolani et al. (2015)
Silk	membrane casting of SF solution	15–30	610–690	4–16	Fenbo et al., 2019, Cao and Wang (2009)
PTFE	100% pure medical-grade bio-inert PTFE	—	4.3	301	Won et al. (2016), Ha et al. (2014), Carbonell et al. (2014)
Magnesium alloys	Smelting ingots casting	41–45	341	7.6	Rahman et al. (2020), Liu et al. (2019)
Titanium and titanium alloys	Selected laser melting	2.34 ± 0.48 (graded porous titanium)	67.63 ± 1.33 (graded porous titanium)	—	Rothamel et al. (2012), Xiong et al. (2020), Zhang et al. (2020)
			2.21–7.85 (3D-printing individualized titanium mesh)		
		110–117 (Titanium alloys)	930–1140 (Titanium alloys)		
Alumina and aluminium alloys	Electrospinning	—	264 ± 4 (coarse-grained alumina)	—	Zhao et al. (2018)
			670 ± 160 (fine-grained alumina)		
			620 ± 40 (ultrafine-grained alumina)		
PCL/PLGA	Solvent casting technique	305.33 ± 65.06	3.48 ± 0.16	—	Kim et al. (2009b), Qian et al. (2016)

**TABLE 3:** Biological properties of barrier membranes.

Material	Cellular cytotoxicity class	Inflammatory reaction	Osteogenic effect	References
PLGA	1	Less macrophages and multi-nucleated giant cells	After 4 weeks, the defects weakly healed	Hoornaert et al. (2016), Li et al. (2016)
		Counts of granulocytes, plasmocytes and lymphocytes, always scored less 2	After 8 weeks, partial bone healing and a few bone spicules were observed	
PLGA/ATT	0	Cell proliferation on the PLGA/ATT was better than that on the PLGA on the 1st, 3rd, and 5th days	More obvious new bone formation, abundant thick bone trabeculae, and significant newly formed capillary vessels in the bone graft area	Xie et al. (2020b)
PCL	0	The presence of PCL further increased the proliferation rate	The PCL mesh infused with bioactive hydrogel facilitated the osteogenic differentiation and mineralization of MSCs	Dubey et al. (2020); Unagolla and Jayasuriya, (2019)
PEG	0	The semi-quantitative histological obsrvation: there was abnormal inflammation, infection or cellular change in the soft tissues	Well-vascularized hard tissue was apparent at all sites	Benic and Hämmerle (2014), Jung et al. (2009b)
			The regenerated bone was similar to the surrounding native bone	
PGA	0	The fibrous connective tissue began to form of the defect after 4 weeks	Histologically, a marked increase in bone formation was observed and bony bridging occurred at 12 weeks. Ct Scans revealed that overall bone regeneration within the defect was achieved at the initial time from 0 to 12 weeks	Lin et al. (2017), Toosi et al. (2019)
Collagen	0 or 1	Patient denied any pain or discomfort	Microscopic examination revealed predominately dense, lamellar bone with scant residual foci of acellular, basophilic graft material	Sbricoli et al. (2020), Açil et al. (2014), Soesilawati et al. (2021), Momen-Heravi et al. (2018)
Chitosan	0	Larger MW (>29.2 kDa) chitosans have anti-inflammatory activity	Autogenous bone showed a histo-morphometric tendency toward increased bone formation during the first month	Oktay et al. (2010), Chang et al. (2019), Ma et al. (2014)
		Smaller MW (≤29.2 kDa) chitosans have pro-inflammatory activity		
Chitosan-collagen	0	No inflammation and residual biomaterial particles were observed on the membrane surface or in the surrounding tissues	The CNC membranes induced significant expression of osteogenic genes in MSCs	Cai et al. (2010), Ma et al. (2016)
Silk	0 or 1	the *in vivo* inflammatory reaction and foreignbody response to silk membranes is similar to or less than collagen membranes	osteoblast-like MG63 cells could attach to, survive on, and proliferated on SF membrane	Yoo et al. (2016), Melke et al. (2016), Meinel et al. (2005)
PTFE	0	An inflammatory tissue reaction within the implantation beds of the PTFE membranes was showed at day 10 post-implantation	The mean bone loss at the proximal and distal margins of the Maxilla was 0.3 and 0.3 mm. The mean bone loss of proximal and distal mandible was-0.2 mm and-0.05 mm, respectively	Chang et al. (2019), Ye et al. (2011), Di carlo et al. (2021)
Magnesium and magnesium alloys	0	No inflammatory response was observed	The experimentally prepared bone defect showed a significant increase in the near distal length 2 months after surgery	Dutta et al. (2020)
Zinc and zinc alloys	0	Intracellular zinc is thereupon free to inhibit IKKβ and negatively regulate the inflammatory process	New bone formed mainly from the periphery of bone defect area to center, and Zn membrane with 300 µm pores manifested evident osteogenic capacity	Lin et al. (2017), Seo et al. (2010)
Titanium and titanium alloys	0	No signs of infection or inflammation appeared	The newly formed ridge dimensions were 6 mm horizontally and 10 mm vertically, with complete filling of the defect observed by CBCT	Amin et al. (2020), Willis et al. (2021), Xie et al. (2020a)
Alumina and aluminium alloys	0	At the highest dosage (50 μm^3^ per cell) there was significant increase in the relative gene expression of IL-8, CCL2, CCL3 and CCL4 in Al_2_O_3_ group	The osteo-immune environment promoted by the 50 nm nano-porous structure was conducive to the osteo-differentiation of BMSCs	Jamieson et al. (2021), Chen et al. (2017)
Novel membranes loaded with drugs or growth factors	0	PRF regulates the inflammatory response, enhances the anti-infection ability, and avoids immune rejection and cross infection	Enamel matrix derivative in a liquid carrier system increased alkaline phosphatase (ALP) mRNA levels 2.5-fold and collagen1alpha2 levels 1.7-fold at 3 days, as well as bone sialoprotein levels twofold at 14 days after inoculation	Miron et al. (2017), Zhang et al. (2017), Shiran et al. (2016), Al-Hamed et al. (2019)
PLGA/PCL	0	hBMSCs were able to proliferate on the porous layer of PLCL bilayer membrane as much as on control membrane though the hBMSCs on the compact layer were significantly less in number than on the control membranes	The highest ALP activity and extracellular calcium deposit were observed on the CS/PCL nanofibrous membrane	He et al. (2017), Abe et al. (2020), Qian et al. (2016)
			The expression of osteocalcin (OCN) and Runx2 were also significantly higher compared to the pure PCL nanofibrous membrane	

### 3.1 Polymer Barrier Membranes

#### 3.1.1 Poly(lactic-co-glycolic acid)(PLGA)

Polylactic acid (PLA) is a novel bio-based and renewable biodegradable material made from renewable plant resources such as straws like cereal husks and starchy crops like maize, sweet potato, and cassava. PLA is a good membrane material for treating bone defects. However, PLA membrane degrades rapidly, and the residue of PLA hydrolysis can cause local inflammation and abscess formation (Ovcharenko et al., 2020). Synthetic polymers of PLA have a higher potential to induce osteogenic differentiation and have been developed as an alternative to natural membranes (Jazayeri et al., 2017). PLGA consists of two monomers, lactic acid and hydroxyacetic acid, randomly polymerized. It is a degradable functional polymeric organic compound with good biocompatibility, non-toxicity, good capsule-forming and membrane-forming properties, which is widely used in pharmaceuticals, medical engineering materials and modern industries. The biodegradable synthetic barrier PLGA membrane generally consists of two layers: a dense layer that prevents invasion of soft tissue cells and the other is a thick microfibrous layer in which the blood clot is stabilized, allowing the blood clot to be stabilized osteoblasts to colonize the membrane. It has been suggested that the degradation rate of PLGA depends on the ratio of the two layers. Its general *in vivo* degradation time is 1–2months (Sun et al., 2017). Hoornaert et al. (2016) demonstrated that the bilayer PLGA membrane maintained structural integrity and barrier function for 16 weeks through *in vitro* and *in vivo* experiments. It was degraded by hydrolysis of polymer chains, which was easy to be applied with artificial bone filling particles for bone defect repair. They concluded that the bilayer PLGA membranes might be a safer and more predictable alternative to GBR. With the developing of PLGA barrier membranes, some composite PLGA material have come into use these years. Attapulgite (ATT) is a kind of natural clay material and an aluminum-magnesium silicate, which is widely distributed in China and the United States. ATT polymer composites have better mechanical durability than the corresponding pure polymers. Moreover, the hydrophilicity of the PLGA fiber membrane is improved with the addition of ATT, which can facilitate the penetration of hydrophilic nutrients and regulate cell response to the membrane surface. Xie X. et al. (2020) confirmed that electrospinning ATT doped PLGA fibrous scaffolds could induce the expression of osteogenic factors in bone marrow mesenchymal stem cells (BMSCs). By effectively promoting bone formation of alveolar bone defects, ATT doped PLGA was demonstrated to be an excellent barrier membrane material.

#### 3.1.2 Polycaprolactone

PCL is a synthetic polymer known for its biocompatibility and excellent mechanical properties. Compared to PLGA, PCL has relatively lower cell affinity. However, with excellent mechanical properties, PCL has the advantage of preventing early fracture of the scaffold. It has been shown that adding silica nanoparticles (Si-NPs) into electrospun PCL membranes could greatly improve the mechanical and osteoconductive properties of the membranes (Castro et al., 2018). With good mechanical properties, PCL can be modified for other barrier membranes. For example, the PCL reticulation prepared by over melt electrowriting can not only delay hydrogel degradation and prevent soft tissue invasion, but also provide mechanical support for the regeneration and differentiation of progenitor cells (Dubey et al., 2020). The bioactivity of PCL was enhanced by mixing it with a natural silk fibroin polymer that has low immunogenicity and inherent bioactivity. It could promote bone regeneration not only in periodontal defects but also in other craniomaxillofacial regions. PCL can be modified by mixing with other materials through electrospinning technology. He et al. (2017) found that chitosan/PCL composite membrane significantly improved the ability of bone formation compared with PCL membrane.

#### 3.1.3 Polyethylene Glycol

PEG is a biodegradable polymer with good biocompatibility. With good processability, PEG can be used as an ideal scaffold material and matrix material for other bone substitutes and blended with other polymers to make various new types of biological barrier membranes (Kim J. Y. et al., 2009; Jung et al., 2015). Wechsler et al. (2008) showed that PEG implantation maintained barrier function for up to 4 months *in vivo*. Some preclinical studies have found that PEG membranes have significant advantages for alveolar ridge bone augmentation in lateral ridge defects (Benic and Hämmerle, 2014). Jung et al. (2009a) used PEG membranes and collagen membranes for regeneration of peri-implant bone defects. They found that the PEG membrane group obtained more bone augmentation and could simplify clinical operation according to the shape of the membrane at the defect site. However, the postoperative exposure rate of PEG membranes (approximately 50%) was much higher than that of collagen membranes (12%) in their study. This may be due to the insufficient antimicrobial properties of the PGE membrane alone. Chitosan-based PEG can be synthesized by photochemical process to improve the thermal stability domain of PGE membrane, so that it has a strong inhibition effect on bacterial growth and reduce the membrane exposure rate (Sautrot-Ba et al., 2019).

#### 3.1.4 Polyglycolic Acid

PGA is an aliphatic polyester polymer material with the least unit carbon count and the fastest degradation rate. Compared with the mainstream like PLA, the current price of PGA is relatively high and its market supply is small. PGA has good biocompatibility, which can be degraded into water and carbon dioxide in the human body, so it is widely used in surgical sutures, fracture internal fixation, tissue engineering repair materials and drug control release system. For its good degradation performance and its degradation product, PGA is rarely applied as a separate barrier membrane but applied by copolymerizating with other materials. The principle is that PGA degrades firstly, leaving pores for the polymer, exposing the active components, increasing the humoral contact area and accelerating the polymer degradation. For instance, biodegradable polymer PGA was blended into the hydroxyapatite/poly-l-lactic acid scaffold fabricated by laser 3D printing to accelerate the degradation (Shuai et al., 2020). A lot of pores produced by the degradation of the scaffold promoted the exposure of HAP from the matrix, which not only activated the deposition of bone like apatite on scaffold but also accelerated apatite growth. Otherwise, the PGA fibers have a significant effect on the physical properties of the collagen scaffolds. *In vitro* cell culture experiments showed that with the incorporation of PGA fibers, the number of attached cells increased higher than that of the collagen sponge (Toosi et al., 2019).

#### 3.1.5 Collagen

Collagen is the main component of extracellular matrix. With particular cell adhesion sites, collagen has excellent properties on regulating cell morphology, adhesion, growth and differentiation. As one of the most widely used barrier membranes (Sbricoli et al., 2020), collagen has good biocompatibility and comes from a wide range of sources (Wessing et al., 2018). Combined with growth factors, collagen membrane is one of the ideal pre-coating membranes, which can induce new bone formation. Compared with Teflon membrane, the surface of collagen membrane is more attractive to osteoblasts and stimulates their proliferation.

Although collagen membrane has good biocompatibility, it still has problems such as irregular degradation, excessive degradation rate, poor spatial stability leading to tissue regeneration damage (Sheikh et al., 2016; Elgali et al., 2017; Tolstunov et al., 2019), lacking osteogenic potential (Zhou L. et al., 2021) and uncontrollable permeability, which can easily cause surgical site collapse (Sheikh et al., 2017b). There are several available methods for improvement. One is the preparation of nanofiber scaffold materials by electrospinning technology, which can obtain good surface area, mechanical strength and biomimetic effect. The material has better performance than polymer fiber (Cai et al., 2010). Second, multi-layer superposition to obtain good mechanical properties. Li et al. (2019) improved the performance of acellular porcine small intestinal submucosa (SIS) by cladding the lyophilized technique. Multi-layer superposition enables the membrane with mechanical solid support. No significant degradation was observed for 4 weeks, while the degradation was complete 12 weeks after implantation, which met the requirements of GBR technology to provide at least 4–6 weeks barrier effect (Moses et al., 2008). Third, collagen cross-linking. Collagen is cross-linked physically or chemically to improve collagen’s mechanical properties and stability used for GBR (Andrei et al., 2017). Fourth, collagen based composite scaffolds for bone regeneration are fabricated by the addition of different biological materials, such as bioceramic, carbon and polymer materials (Zhang et al., 2017). It has also been suggested that by concentrating collagen membranes, the barrier functions for up to 90 days. But it may lead to altered patterns of inflammatory tissue response (Gueldenpfennig et al., 2020).

Acellular dermal matrix (ADM) membrane is also a kind of collagen membrane. After removing cells in autologous or allogeneic skin tissue, the extracellular matrix with complete structure is retained, which can be used in repairing cartilage defects with type II collagen composite modification (Tiruvannamalai Annamalai et al., 2016). Studies have found that acellular matrix membrane can further promote the production of osteogenic chemokines by forming a local microenvironment conducive to the rapid recruitment of mesenchymal stem cells during bone remodeling. For example, C-X-C chemokine receptor type 4 (CXCR4) and monocyte chemoattractant protein-1 (MCP-1), etc. (Siddiqui et al., 2017; Jin et al., 2018). Momen-Heravi et al. (2018) applied ADM to repair peri-implant bone-cracking and found that new bone was formed around the bone defect area with fine effect of bone reconstruction.

Human amniotic membrane (HAM) is one of the oldest biomaterials used for wound healing in medicine, which consists of collagen and glycosaminoglycan (GAG) as main structural components (Rameshbabu et al., 2016). Similarly to HAM, chorionic membrane is four to five times thicker than HAM (Koob et al., 2015). HAM is a good source of growth factors (Grzywocz et al., 2014), and it is considered as a surgical waste (Fénelon et al., 2018), so it is a highly available and cost-efficient tissue that might be advantageous for bone regeneration. After conservation of extracellular matrix components in fresh and preserved HAM, Fenelon M et al. found that decellularization/lyophilization of HAM increased its mechanical properties and enhanced osteogenic differentiation of human bone marrow stromal cells (hBMSCs), which significantly increased early bone regeneration (Fenelon et al., 2020). Ghanmi et al. found that fresh acellular HAM promoted bone regeneration at the critical size of bone defect (Ghanmi et al., 2018). Besides creating a decellularized amnion/chorion membrane to improve the stiffness of this membrane (Koob et al., 2015), another possibility expected to further enhance the mechanical properties of HAM is to design a multilayered DL-HAM scaffold (Swim et al., 2019).

#### 3.1.6 Chitosan

Chitosan, also known as deacetylchitin, is chemically known as polyglucosamine (1-4)-2-amino-B-D glucose. Chitosan has been widely used in bone tissue engineering in recent years because of its good permeability, antibacterial and immunomodulatory activity, and procoagulant wound healing properties (Ma et al., 2016; Tavelli et al., 2020). The porous scaffolds of chitosan reinforced with calcium phosphate can be used as a bone substitute for bone repair and dental fillings. Besides, chitosan can be degraded by lysozyme in organisms to generate natural metabolites, which are non-toxic and can be absorbed entirely by organisms. Therefore, it is superior to be used as a drug sustained-release agent and applied in controlled release system of drug and growth factors (Morris et al., 2010). Chitosan membrane was implanted subcutaneously in rats and was found to have the ability of maintaining barrier function for up to 6 weeks (Xu et al., 2012). The degradation rate, as well as the mechanical properties of chitosan membranes can be improved by changing their molecular weight and preparation methods. By electrostatic spinning technique, chitosan can be prepared into biofunctional composite with more rigid materials.

#### 3.1.7 Chitosan-Collagen

The addition of chitosan to collagen scaffold material can change the cross-linking of collagen fibers and enhance the structure of the composite material. The scaffold has good bone conductivity, mechanical strength and biological stability (Martino et al., 2005). Collagen electrospun nanofiber is superior to a solid wall in cell proliferation and differentiation. Therefore, chitosan reinforced collagen nanofiber membrane can be used as a natural biological composite polymer to prepare bioabsorbable membrane for GBR purposes. *In vivo* experiments showed that the fibrous structure of chitosan-collagen nanofibrous scaffold material membrane improved the osteogenic differentiation of bone marrow mesenchymal stem cells (MSCs). Lotfi et al. (2016) developed a chitosan-collagen nanofiber scaffold material. The marginal bone maturation effect was better than that of the bio-guide membrane covered group, with normal inflammatory response, indicating good bone conduction, mechanical strength and biocompatibility. Chitosan has osteogenic properties (Thein et al., 2008) and good tissue separation (Bumgardner et al., 2007). It also has a specific modification effect on collagen biomechanical properties.

#### 3.1.8 Silk

Silk, a macromolecule produced by Cocoon (*Bombyx mori*) or Spider (*Nephila clavipes*), can be made into a variety of biomaterials, including membranes, porous scaffolders, gels, suture materials and non-woven webs after purified (Liu et al., 2013; Qi et al., 2017). It is worth mentioning that silk fibroin (SF), a structural protein of silk, has high biocompatibility and less foreign body reaction (Midha et al., 2016; Saleem et al., 2020; Luo et al., 2021). Compared with collagen membrane, SF, as a new biomedical material, has good biocompatibility as well as excellent mechanical properties, controllable degradation and plasticity, which has been widely used in the research of bone tissue engineering. Lee et al. (2014) prepared a thin silk membrane with fiber network structure by a simple separation method. The tensile strength of the silk membrane is similar to that of the collagen membrane in dry state, while higher than that of the collagen membrane in wet state. It was found that SF membranes showed good bone regeneration in the skull defect model with less inflammation and similar volume of bone regeneration was observed compared with collagen membranes (Kim et al., 2014; Song et al., 2014). Yoo et al. (2016) proved that osteoblast-like MG63 cells could attach to, survive on, and proliferated on SF membrane, as a hint of its suitableness for the bone regeneration process. Due to the low cost of SF membranes and zero risk of infection transmission from animal tissues, their clinical use should be encouraged as an alternative to collagen membranes widely used in GBR.

#### 3.1.9 Alginate

Alginate is salt derivatives of alginic acid and constitute long chains of polysaccharides, which provides pliability and gelling adeptness to their structure. It has favorable biocompatibility and can be cross-linked to form hydrogels to form structures similar to the extracellular matrix. It can be coated directly on the surface of the bone defect area. The alginate membrane provides a sound barrier between the connective tissue and the bone defect area. The main defect of alginate barrier membrane is its poor mechanical properties. The membrane often collapses towards the bone defect area, leading to the reduction of osteogenic effect. Therefore, alginate is suitable to be used with other materials with particular strength to prepare composite membranes or improve processing technology to overcome its shortcomings further. Take an effective interaction between the sodium alginate chain and the nanosized hydroxyapatite for an example (D'Elía et al., 2020). Higher nanoscale hydroxyapatite concentrations increase the length and branching of the polymer network, which in turn reduces the water content in the structure. The viscosity and strength of fresh hydrogels are increased while the plasticity of the membranes was reduced.

#### 3.1.10 Poly Tetra Fluoroethylene

PTFE is a macromolecular compound, which is one of the most widely used polymers in polymer non-absorbable barrier membranes. PTFE membrane has good biocompatibility and can effectively protect blood clots, so it is regarded as the gold standard of barrier function materials (Jazayeri et al., 2017). PTFE membrane should ensure good tissue tightness in the treatment process. Expanded PTFE (e-PTFE) is a polytetrafluoroethylene treated by expansion or stretching, which has the sealing property of polytetrafluoroethylene. At the same time, the plastic deformation of modified polytetrafluoroethylene occurs under long-term continuous load. However, bacteria are easy to invade through the pores of the membrane and cause postoperative complications. If it is exposed to the human oral environment before the end of treatment, it will lead to treatment failure. Due to the occurrence of postoperative complications of membrane exposure caused by its easy bacterial invasion, it is gradually replaced by a smaller pore size Dense PTFE (d-PTFE) membrane. But, because the porosity of the d-PTFE membrane is limited, so the blood supply to this area is limited. Commercially available non-resorbable barrier membranes are usually made of Teflon. It is biocompatible, which maintains its integrity during and after implantation. It can not only treat significant, drug-free bone defects and multiwall defects, but also can affect vertical enhancement (Caballé-Serrano et al., 2018). Different from the previous view that PTEE is a bioinert material, Korzinskas et al. (2018) found that the severity of proinflammatory and anti-inflammatory PTEE was equivalent to that of collagen membrane with good biocompatibility. It is not wholly a bioinert material, which can best support bone healing in the context of inducing bone regeneration.

#### 3.1.11 Other Polymers

Some other polymers can also be used in the fabrication of barrier membranes. Hyaluronic acid (HA), a polyanionic natural polymer, is one of the main components of the hydrophilic polymer and extracellular matrix (Sudha and Rose, 2019). Hyaluronic acid membranes were widely used in the field of orthopedics due to the effectiveness in wound healing. Studies have indicated that HA membrane has an effect close to that of the collagen (Yilmaz et al., 2021). Furthermore, membranes based on HA derivatives hold great potential to stimulate mineralization for the periodontal regeneration (Federico et al., 2021), which can be investigated for periodontal barrier applications and used to achieve bone regeneration (Park et al., 2009). Ayanoğlu et al. (2015) conducted a study on the combined use of HA membrane and allograft on bone defects of rabbits. Results indicated that the use of HA membranes alone or in combination with graft can promote bone healing.

Poly(3-hydroxybutyrate) (PHB) is a linear of naturally produced polyesters, unbranched homopolymer consisting of (R)-3-hydroxybutyric acid units, which stored in cells by prokaryotic microorganisms under the condition of nutritional imbalance (Valappil et al., 2007). It also has the characteristics of natural degradable materials and the advantages of synthetic degradable materials (Rodriguez-Contreras, 2019). Karahaliloğlu et al. (2015) fabricated NaOH treated PHB membranes, which showed increased proliferation of human osteoblasts for GBR applications. Partial bone regeneration can be observed in bifurcation defects in dogs by the application of rigid absorbable membranes made of hydroxyapatite and PHB (Reis et al., 2013). In addition, PHB not only degrades into non-toxic oligomers (Chiulan et al., 2017), but also has been found to have positive effects on mesenchymal stem cells (Voinova et al., 2019), which might be a suitable candidate for *in vivo* use in medical applications.

### 3.2 Non-Polymer Barrier Membranes

#### 3.2.1 Magnesium and Magnesium Alloys

Compared with other orthopedic degradable materials, the local magnesium-rich environment generated by the degradation of magnesium-based materials can activate different signaling pathways through various signal mediations. The environment can stimulate bone regeneration, improve the adhesion rate of osteoblasts, inhibit the activity of osteoclasts and regulate the signal transduction of osteogenic cells. Magnesium alloy has relatively active chemical properties and is easily degraded and absorbed in the physiological environment. Its degradation can promote calcium deposition. The rate of osteogenesis is fast in the early stage of osteogenesis. Its bone-guiding property is better than titanium as well (Rahman et al., 2020). In addition, magnesium has Young’s modulus similar to that of human bone, which can avoid stress shielding. Thereby the surrounding natural bone can be transferred to the defect and stimulating bone remodeling (Dutta et al., 2020). Different from titanium, magnesium alloy products can self-degrade after bone tissue repair and regeneration. It can promote bone tissue healing because magnesium ion is an essential element for the human body, which is convenient for clinical promotion. In addition, magnesium alloy does not interfere with CT and MRI imaging examination after surgery (Farraro et al., 2014), which facilitates the effect after implantation. Si et al. (2021) have studied a Mg-2.0Zn-1.0Gd alloy (wt%, MZG) membrane for biomedical use. The alloy material has a favorable plastic deformation ability. In order to improve the biological corrosion resistance and cytotoxicity *in vitro*, a biodegradable magnesium alloy GBR Mesh plate with 0.6 mm Ca-P coating was developed. Experiments indicate that MZG has excellent potential for clinical application as GBR membranes.

The degradation rates of magnesium alloy are too fast and the degradation *in vivo* produces hydrogen evolution reaction, causing increase in local PH. It affects the growth of the surrounding tissue, hemolysis, dissolved bone happens, even severely limited the application of magnesium alloy in clinic. Modification of magnesium alloys can be achieved by adding alloying elements as well as surface modification. Physical modification, such as modifying microstructural features, can effectively slow down the degradation of magnesium alloy *in vitro* and *in vivo*, reduce local hydrogen evolution and pH raise, which ulteriorly improves its biocompatibility (Wu et al., 2019; Saberi et al., 2021). Magnesium alloys can be modified by integrating with other materials such as collagen. Barbeck et al. (2020) embedded HF-treated magnesium-based meshes in a collagen membrane in order to combine the mechanical properties of degradable metal with the biocompatibility of collagen membrane. However, Steigmann et al. (2020) believed that even if the integration behavior of collagen membrane was comparable, pure magnesium membrane could still meet the requirements of biocompatibility.

#### 3.2.2 Zinc and Zinc Alloys

As an essential trace element, Zinc involves in a variety of essential biological functions such as nucleic acid metabolism, signal transduction, apoptosis regulation and gene expression (Dermience et al., 2015; Lin et al., 2017). An vitro study showed that zinc degradation in saline solution did not have a large effect on the surrounding cultures and that the zinc corrosion rate was accelerated after 120 h due to passivation (Meng et al., 2018). Zinc is a clinically ideal material with an intermediate degradation rate between magnesium and iron. Zinc plays a crucial role in the growth and mineralization of bone tissue, which directly activates aminoacyl tRNA synthetase in osteoblasts (Seo et al., 2010) and stimulates cellular protein synthesis (Storrie and Stupp, 2005). In addition, zinc inhibits bone resorption by inhibiting the formation of osteoclast-like cells in bone marrow cells (Frederickson et al., 2005). Zinc also plays an essential role in the preservation of bone mass (Amin et al., 2020). Guo et al. (2020) concluded that microporous pure zinc membranes of 300 μm have superior MC3T3-E1 cytocompatibility *in vitro* and osteogenic capacity *in vivo*, which have potential bone regeneration applications in GBR membranes.

#### 3.2.3 Titanium and Titanium Alloys

As a non-resorbable metal barrier membrane, titanium mesh can be used as a barrier membrane alone. It can also be used to strengthen absorbable collagen membrane and enhance polytetrafluoroethylene, etc. Good biomechanical properties of titanium membrane (Alagl and Madi, 2018) can effectively maintain osteogenic space and prevent the migration of epithelial and connective tissue cells. It has been widely used to reconstruct significant jaw defects and alveolar bone defects (Abdel-Hady Gepreel and Niinomi, 2012). Bai et al. (2019) found through 3D printing technology that the thickness and aperture of titanium mesh affected the amount of new bone under the titanium mesh. Appropriate pore size and thickness played a crucial role in promoting the growth of bone tissue. It has been concluded that 0.4 mm thickness titanium mesh not only carries enough strength but also has less stimulation to the mucosa and is more suitable for clinical use. Alagl and Madi (2018) combined a nano-bone graft and alloplastic with Ti-mesh for local Ridge Augmentation. It seems to be a clinically feasible method to repair soft tissue and complex tissue defects in a relatively short time so that suitable implants can be implanted in a short period.

#### 3.2.4 Alumina and Aluminium Alloys

In physiological environment, alumina is an inert material matrix, which is non-absorbable and extremely hard. Al_2_O_3_ appeared to have no significant cytotoxic effect (Jamieson et al., 2021), however, aluminium is not considered to exhibit osteogenic properties and even recognized for having adverse effects on osteoblasts (Yang et al., 2018). To overcome these limitations, some composites have been developed from two or more materials to obtain the excellent properties of a single material. For instance, bioglass and hydroxyapatite-coated porous alumina scaffolds showed higher biocompatibility and osteointegration responses compared with pure alumina scaffolds (Camilo et al., 2017). In fact, alumina is more commonly used as a sandblasting coating in dentistry. The use of particles of different sizes to obtain regular roughness values results in changes in osteoblast behavior and binding to bone (Kim et al., 2006). It also stimulates calcium to flow out of the bones (Bushinsky et al., 1995). Studies showed that titanium aluminium vanadium (TiAIV) implants sandblasted with Al_2_O_3_ showed higher stability ratio (ISQ) and removal torque values than TiO_2_ and SiO_2_ at the end of the first and third months, and had a better effect on bone bonding (Yurttutan and Keskin, 2018).

## 4 Functional Membranes

With the development of barrier membrane, absorbable functional membrane has received increasing attention these years. There are three main construction methods: 1) Loading antibacterial materials to reduce the repair failure rate caused by inflammation; 2) Loading bioactive factors to increase bone mass through their own osteogenesis and synergistic osteogenesis; 3) The multilayer structure is manufactured to meet different requirements of different contact surfaces such as implant surface, bone surface and epithelial tissue in bone regeneration.

### 4.1 Antibacterial Barrier Membranes

When loaded with antibiotics, growth factors and adhesion factors, the synthetic membrane can be used as a delivery device for specific preparations. To ensure the effectiveness of guided bone regeneration, it is necessary to reduce inflammatory response caused by bacterial invasion. First, antibiotics were added to the barrier membrane surface. Antibiotics in the barrier membrane can effectively reduce the risk of postoperative infection. Moreover, antibiotics can slow down collagen degradation. There are many antibacterial materials can be loaded onto the barrier membranes. PEG, superhydrophobic structural coating, biomembrane matrix-degrading enzyme coating, silver-releasing ion coating, titanium dioxide photoactive coating, chlorhexidine and other substances are widely used in the fabrication of antibacterial barrier membranes. Second, the application of structural drug-loaded sustained-release systems. At present, electrospinning technology, as a traditional textile industry applying polymer technology, is introduced as a novel production strategy for nanomimetic scaffolds in the field of tissue engineering. It is also a promising controllable drug delivery system, which allows the addition of therapeutic agents to the mesh eye of nonwoven nanofibers during electrospinning. Polymer nanoparticles, nanotubes, micelles, and lipid nanoparticles can be combined with electrospun nanofibers to improve the release profile, drug safety, loading efficiency and better functionality of the fibers. The commonly used therapeutic agents are aspirin (Ghavimi et al., 2020), azithromycin (Wang Z. et al., 2019), metronidazole (He et al., 2018), antibacterial peptide (Wei et al., 2018) and so on. In addition, composite encapsulating antibacterial agents can be prepared by cross-linking technology and may even be used as drug carriers in pH-responsive controlled release drug delivery systems (Mathew et al., 2017; Bi et al., 2019).

### 4.2 Bioactive Barrier Membranes

The main research hotspot of modern tissue engineered bone construction is how to control a variety of growth factors with different biological activities in time to play a role in different stages of bone healing and mimic the natural osteogenesis process. It is currently proved that the most important factors are osteogenic factors and angiogenic factors. In recent years, the commonly used pro-bone tissue growth factors include bone morphogenetic protein (BMP), vascular endothelial growth factor (VEGF), platelet-derived growth factor (PDGF), insulin-like growth factor (IGF), and transforming growth factor (TGF). BMP is one of the most widely used and studied growth factors for bone tissue engineering. During bone remodeling, signal analysis of the temporal and spatial expression of BMP genes showed that they were very similar to osteogenesis and fracture healing. BMP-2 is one of the relatively known bone formation effects among all growth factors, which has the ability to promote the directional differentiation and proliferation of mesenchymal stem cells into osteoblasts. It can be combined with a variety of carriers with different properties to compose various types of bioactive restorative materials. BMP-loaded carriers can be supramolecular hydrogels (Tan et al., 2019), core-shell nanofibers (da Silva et al., 2019), acellular collagen sponges (Sun T. et al., 2018), etc. The BMP-loaded barrier membrane can obtain site preservation after tooth extraction and obtain bone enhancement at the bone defect site. Among them, the amount of bone enhancement was proportional to the dose of recombinant human BMP-2 (rhBMP-2) (Sun Y.-K. et al., 2018). Thoma et al. (2018) demonstrated that rhBMP-2-loaded xenograft bone blocks showed little difference in results at 4 months compared with autologous bone blocks that were considered as the gold standard, though autologous bone blocks were more mineralized. However, in recent years, rhBMP-9 has shown better osteogenic differentiation ability compared with rhBMP-2 (Fujioka-Kobayashi et al., 2017). Another important growth factor, VEGF, is considered to be the most potent growth factor known to induce angiogenesis (Zhang et al., 2021). It not only affects the differentiation of osteoblasts, but also plays an important role in cartilage resorption and promoting the early and late chondroossification of angiogenesis. The combined use of VEGF and rhBMP-2 can increase angiogenesis and blood supply, promote the formation of new bone and solve the problem of vertical bone regeneration in clinical work (Schorn et al., 2017). There is also TGF (Tan et al., 2021) similar to BMP synergistic osteogenesis. In addition to growth factors, some animal and plant active components such as BMP-2-related peptide P28 (Sun T. et al., 2018), amelogenin (EMD) (Miron et al., 2017) and Icariin (ICA) (Yin et al., 2017b) have also been applied in bone tissue engineering.

### 4.3 Structurally Layered Barrier Membranes

Multilayer barrier membranes have been designed to meet more complex requirements to endow the barrier membranes with more affluent properties. However, although the membranes are divided into multiple layers, they should still overall meet the primary purpose of GBR, so each layer should have different biological characteristics. For example, the pore size of the inner layer (loose layer) should be large enough to meet the specific requirements of the contact surface between barrier membrane and epithelial tissue, which can stabilize the blood clot and promote the integration of soft tissue in a double-layer membrane. The outer layer (dense layer) has a relatively small pore size to maintain the osteogenic space, block the entry of soft tissue cells in the same time and allow the stem cells and nutrients to pass through. In addition, there is a gap between the bilayer structure to facilitate tissue integration (Rothamel et al., 2012). Kim S.-H. et al. (2009) prepared a double-layer collagen membrane and implanted them in rats. They found that compared with the monolayer collagen membrane, the non-crosslinked collagen membrane bilayer technique could improve the bone resorption and bone strengthening effect of the embedded bone grafting technique. Tai et al. (2014) prepared and found a polyhydroxybutyrate-biphasic calcium phosphate/chitosan membrane that had the role of guiding bone regeneration barrier membranes in periodontal tissue engineering. The membrane developed by Sheikh et al. (2016) had one surface of polyether urethane (PEU), while the other side was hydroxy-terminated polydimethylsiloxane (PDMS) coated with PEU. The non-PDMS coated side adsorbed proteins, which could repair defects in periodontal tissue regeneration and attract growth factors, while the PDMS coated side acted as a barrier for gingival epithelial cells to prevent proliferation and migration of gingival epithelial cells into the defect gap through the soft tissue flap. PCL/PLGA scaffolds obtained biological and mechanical advantages during the mixing process, making up for their respective shortcomings (Kim J. Y. et al., 2009). Abe et al. (2020) made a double phase-out membrane by solid. The PLGA and PCL polymerization method of controlling the membrane degradation can reduce the cost of placing two or more than two membranes in the clinic and reduce the waste of membrane barrier materials.

## 5 Common Processing Techniques for Barrier Membranes

The barrier membrane processing techniques include electrostatic spinning (Yin Y. et al., 2017), 3D printing (Bai et al., 2019), chemical crosslinking (Oryan et al., 2018), phase inversion (Fu et al., 2017) and other technologies. All kinds of preparation methods have different characteristics. Among them, electrospinning and 3D printing are the most widely used preparation technologies.

Electrospinning technology refers to a method in which polymer solution or melt produce jet stretching under the action of high-pressure electrostatic field force to obtain ultrafine fiber by solvent volatilization or melt curing. This technique can be used to prepare natural polymers such as collagen, SF, alginate; polymers such as PLGA, PCL, PEG, PGA, PTFE; inorganic materials such as alumina, zinc oxide; composite materials such as chitosan-collagen. The nanofibrous film it prepared has a very large specific surface area and a very high porosity (Henry et al., 2017). Compared with traditional materials, electrospun nanofiber scaffolds not only have good diversity and high parameter controllability, but also have unique advantages in personalized applications under different tissues and physiological conditions (Zhang et al., 2012). However, it is still difficult to conduct large-scale nanofiber production because of the slow electrospinning speed, low yield, and nanofiber with too small diameter cannot be obtained. The industrialization research of electrospinning industry should be strengthened in the future.

3D printing is a kind of fast forming technology, based on mathematical model documents (Trenfield et al., 2019). It uses metal powder, plastic and other adhesive materials to make products through heat sources such as laser. 3D printed granular bone graft could better maintain bone defects and support barrier membrane without corresponding clinical tests instead. The design of 3D printed custom titanium mesh avoids nerves and blood vessels, which is important for improving the precise reconstruction of GBR and providing enough space for implantation to reduce exposure rates (Zhou T. et al., 2021). In addition, 3D printing technology is also promising for polymers (Hwang et al., 2017). Park (Park et al., 2018) used 3D printing technology to print a three-dimensional PCL stent adapted to the bone defect area of the animal model and implanted it with β-tricalcium phosphate (β-TCP) powder which successfully maintained the physical space of the bone defect site and promoted the postoperative regeneration of healthy bone without an inflammatory or infectious response. However, while 3D printing solves the needs of personalization and precision in implantation, there are still some problems, such as higher cost, more complicated individual printing process and still deviation, etc.

## 6 Prospects of the GBR Technique

There are many shortcomings in current GBR technology. First, barrier failure may occur when applying membranes. One scenario is that the membrane is exposed to the oral environment due to poor soft tissue closure. Another circumstance is that the soft tissue is well sealed but the absorbable membrane degrades and loses the barrier protection function. Moreover, it relies mainly on the body’s growth potential to repair the defect. If the activity and number of stem cells are insufficient, it will difficult to predict the osteogenic effect. Therefore, it is difficult to gain satisfactory osteogenic results, if the defect is large or the body is in poor condition.

With the fast development of GBR, further improvement on barrier membrane will become to one of the most important fields in bone regeneration. One is to improve the physical and chemical properties of the membrane to enhance the barrier function, such as the use of new processing methods, preparation of multilayer structure. The other is try to improve the ability of osteogenesis, such as loading drugs and various growth factors, combining osteogenesis and angiogenesis, further reducing the immune response and so on. By enhancing the use of growth factors in the barrier membrane, GBR will improve bone regeneration effects. If the bone mass and bone are stable, it is prospective that GBR could be extended to improve bone augmentation outside the mouth. For example, GBR may become the normally auxiliary means for repairing systemic tissue defect, such as the non-benign bone defect of diabetes and osteoporosis patients, even more extensive defect repair after injury.
